# Impact of surgeons’ experience and the single-shot perioperative antibiotic prophylaxis on outcome in stapedotomy

**DOI:** 10.1371/journal.pone.0247451

**Published:** 2021-02-23

**Authors:** Faris F. Brkic, Boban M. Erovic, Arina Onoprienko, Stefan Janik, Dominik Riss, Claudia Lill, Stefan Grasl, Jafar-Sasan Hamzavi, Erich Vyskocil

**Affiliations:** 1 Department of Otorhinolaryngology, Head and Neck Surgery, Medical University of Vienna, Vienna, Austria; 2 Institute of Head and Neck Diseases, Evangelical Hospital Vienna, Vienna, Austria; Universidade Federal de Sao Paulo/Escola Paulista de Medicina (Unifesp/epm), BRAZIL

## Abstract

**Background:**

The aim of this study was to evaluate whether surgeons´ experience and perioperative single-shot antibiotic prophylaxis affect outcome of patients undergoing stapes surgery.

**Patients and methods:**

We retrospectively evaluated audiological outcomes and postoperative complications of 538 consecutive patients who underwent stapes surgery at a single tertiary referral center between 1990 and 2017. Effects of different clinical variables, including single-shot antibiotic prophylaxis and surgeons’ experience on outcome were assessed.

**Results:**

538 patients underwent 667 stapedotomies and postoperative complication rate was 7.5% (n = 50). Air conduction and air-bone gap closure improved significantly after surgery (14.2 ± 14.8 dB, p = 0.001; 14.5 ± 12.8 dB, p = 0.001). Multivariate analysis revealed that 6 years or less of surgical experience was independently associated with a higher incidence of persisting or recurrent conductive hearing loss (p = 0.033, OR 5.13) but perioperative application of antibiotics had no significant effect on outcome.

**Conclusion:**

First, clinical outcome regarding persisting or recurrent conductive hearing loss caused by incus necrosis and prosthesis luxation is linked to surgical performance. This underlines the need for a meticulous training and supervision of less experienced surgeons performing stapes surgery. Second, our results do not support the need for perioperative antibiotic prophylaxis in stapes surgery. Potential standard limitations of retrospective cohort studies (selection bias, confusion bias etc.) could play a role in interpreting our results. However, the probability for these limitations is minimized due to the large patient sample.

## Introduction

Besides conductive hearing loss, many patients with otosclerosis develop in particular a sensorineural hearing loss without association to age-related hearing loss [1]. Therapy options include hearing aids and stapes surgery [2]. Stapes surgery involves removing the stapes superstructure and replacing it with a prosthesis. It is proven to be a safe and low-risk procedure [3]. However, there is a risk of permanent postoperative complete sensorineural hearing loss in approximately 1% of patients [4]. Further complications include postoperative vertigo, tinnitus, infections and luxation of the prosthesis [5]. Audiological outcomes of stapes surgery are usually assessed by improvement in air conduction (AC), in pure field pure-tone audiometry (PTA) and the air-bone gap (ABG) closure [6, 7].

The impact of antibiotics in ear surgery has been discussed in the last decades. One study group noted that no perioperative antibiotic prophylaxis is needed in clean head and neck surgical procedures [8]. In contrast, a significantly lower complication rate was observed in surgery on contaminated cholesteatoma where a perioperative antibiotic single-shot was administered [9]. Regarding reports on clean otologic surgery, literature is sparse and fairly outdated. Two reviews of non-recent literature from earlier decades recommend no need for antibiotic prophylaxis in clean otologic surgery (including stapes surgery) [10, 11]. However, there is no clear evidence regarding perioperative antibiotic treatment exclusively in stapes surgery.

Furthermore, the association between the level of surgical experience and surgical outcome in stapes surgery has not been yet addressed by literature even though this question has been raised for other surgical procedures [12–14].

Therefore, the present study aims to analyze outcomes and postoperative complications of stapes surgery in a single tertiary referral center over a period of 27-years. We evaluate the impact of the surgeon’s experience and the perioperative application of antibiotics on audiological outcome and on postoperative complications.

## Material and methods

### Study cohort

This retrospective study was conducted at a tertiary academic referral center (Department of Otorhinolaryngology, Head and Neck Surgery at the Medical University of Vienna, Austria). All patients treated with stapes surgery with available follow-up data were included in the study.

### Clinical data

Data on inpatient and outpatient activity of all included patients were extracted from the Vienna General Hospital database system (AKIM). All patients undergoing stapes surgery between 1990 and 2017 were included. Subjective audiologic testing was performed with routinely calibrated audiometers and included SF PTA pre- and postoperative bone- and air-conduction thresholds (at 500, 1000, 2000, 3000 and 4000 Hz). Furthermore, data on age at the time of surgery, sex, date of surgery, perioperative single shot antibiotic (yes/no), perioperative single shot cortisone (yes/no), use of laser (yes/no), prosthesis type, experience of the surgeon (years) and postoperative complications were analyzed. The years of experience were counted from the first surgery that was performed unsupervised. Audiological outcomes included the ABG closure and the AC improvement in pure-tone PTA and effects of above-mentioned parameters on the outcomes were tested. Postoperative complications included severe vertigo (2^nd^ or 3^rd^ grade nystagmus with nausea persisting for more than three days), nausea, taste disturbance, wound infections or dehiscence, sensorineural hearing loss including total hearing loss (>10 dB) and conductive hearing loss.

### Statistical methods

Statistical analysis was performed using Statistical Program of Social Sciences (SPSS, version 23.0; SPSS, Inc., Chicago, Ill). Statistical significance was set at 0.05, two-sided. Due to the large patient sample, a normal distribution could be assumed. Therefore, mean and standard deviations were used for descriptive analysis. In order to determine statistical significance, chi-square, paired and unpaired t-test and spearman correlation were used. Furthermore, a binary logistic model was built. Metric variables were dichotomized according to the median value. Variables tested at p < 0.05 in the univariable analysis were included in the multivariable model.

### Ethics

This study was conducted in compliance with Helsinki Declaration and was approved by the institutional research board of the Medical University of Vienna (EK1215/2017). Patient data was partly anonymized (“pseudonymization”—each patient was assigned a code number). Therefore, based on the guidelines of the institutions ethical committee, no informed consent was needed.

## Results

### Patient cohort

Between 1990 and 2017, 538 patients with otosclerosis underwent 667 stapes surgeries, including bilateral and revision procedures. Each procedure was analyzed separately, since many patients underwent bilateral and revision surgeries. Mean age of patients at the time of surgery was 43.6 years (range 13.2–87.7 years, median 43.9 years). 362 patients were female (63.3%) and 176 male (36.7%). A PTA across frequencies (500 to 4000 Hz) as well as temporal bone computer tomography scan was performed in all patients preoperatively.

### Surgery

Perioperatively, an antibiotic single-shot was applied in 364 (54.6%) and corticosteroids were administered in 513 surgeries (76.9%). All ears were operated under general anesthesia. A stapedotomy technique was used in all patients via an endaural approach with lifting of the tympanomeatal flap. In 208 cases (31.2%), cutting of the posterior crus and perforation of the footplate was performed with a CO_2_ laser. A gold piston was used in 366 surgeries (54.9%), a platin-teflon prosthesis in 230 (34.5%), a titanium prosthesis in 44 cases (6.6%) and 27 ears were provided with other implant types (4.0%). After crimping of the prosthesis, the mobility of the ossicles and the light reflex of the round window was controlled. The surgery was finished by reposition of the flap, tamponade in the external auditory canal and surgical wound closure.

The average experience of the surgeon at time of the surgery was 7.0 years (range 1.0–27.0 years, median 6.0 years). Postoperative complications occurred in 50 cases (7.5%) and are listed in Table 1. A significant postoperative sensorineural hearing loss (>10 dB) was observed in 15 (2.2%) patients. Notably, a surgical site infection (SSI) was noted in only one (0.1%) patient. Moreover, 1 (0.1%) patient developed severe postoperative vertigo (with nausea and persisting 3^rd^ grade nystagmus). A surgical revision and an explanation of the stapes prothesis was performed 3 weeks after surgery. In a total of 15 cases (2.2%), a recurrent or persisting postoperative conductive hearing loss was observed Eight of these underwent a surgical revision in the follow-up. Interestingly, intraoperative findings revealed prosthesis luxation and necrosis of the long crus of incus in 6 and 2 patients, respectively.

**Table 1:** Postoperative complications.

Complication	n	%	revision,n
Perceptive hearing loss (>10 dB)	15	2.2	4
Persisting or recurrent conductive hearing loss	15	2.2	8
Vertigo	14	2.1	1
Wound dehiscence	1	0.1	0
Middle ear fistula	1	0.1	1
Eardrum perforation	1	0.1	1
Granuloma in outer ear canal	1	0.1	1
Wound infection	1	0.1	0
Severe taste disturbance	1	0.1	0
Total	50	7.5	16

Regarding audiological outcomes, the average postoperative AC-improvement of 14.2 +/- 14.8 dB was observed in all patients and the mean ABG-closure was 14.5 +/- 12.8 dB.

### Outcome

**Fig 1:**
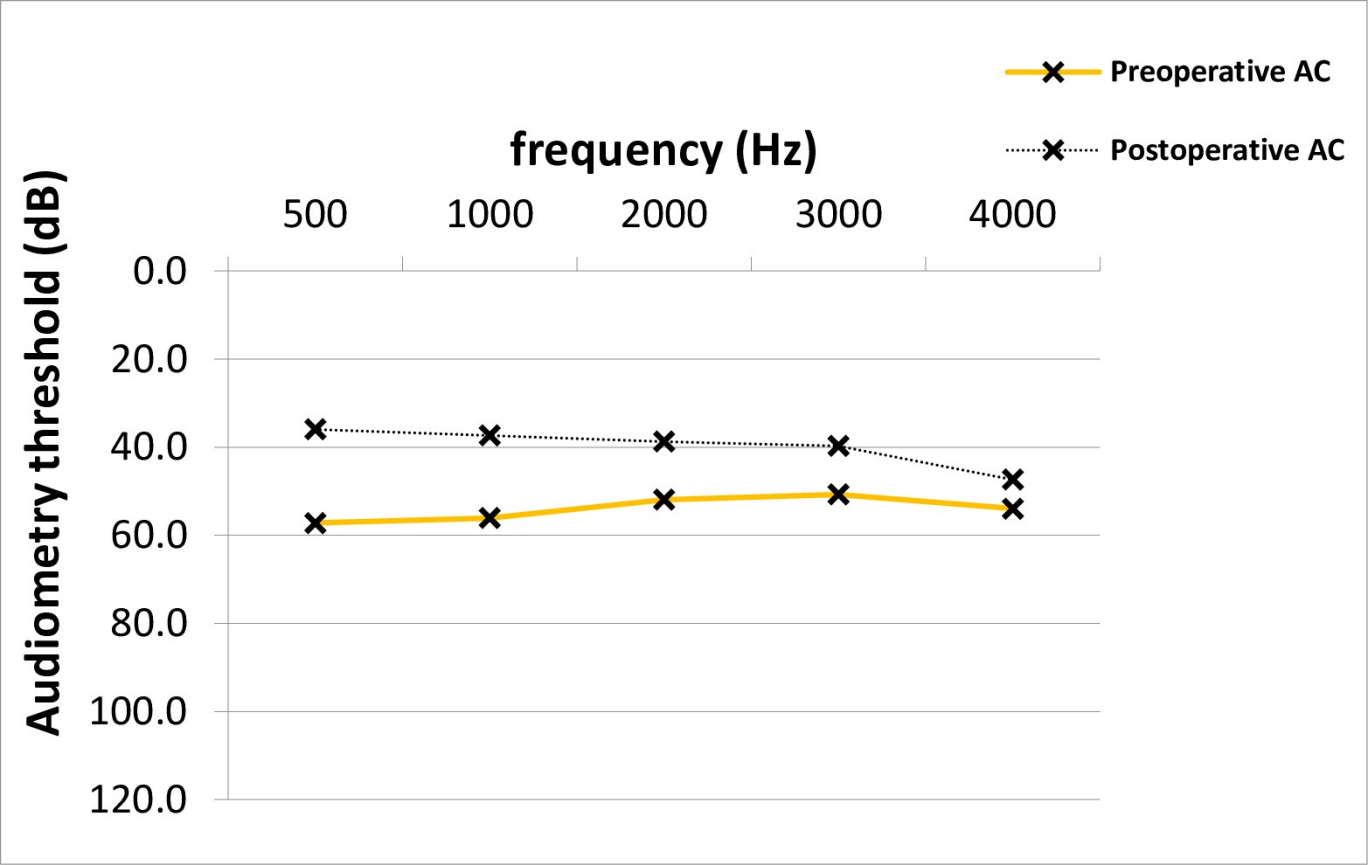
AC Improvement across frequencies in PTA. The pre- and postoperative average AC values across frequencies differed statistically significantly (Paired T-test, p = 0.001). AC; air-conduction, PTA; pure-tone audiometry, dB; Decibel, Hz; Hertz.

**Fig 2:**
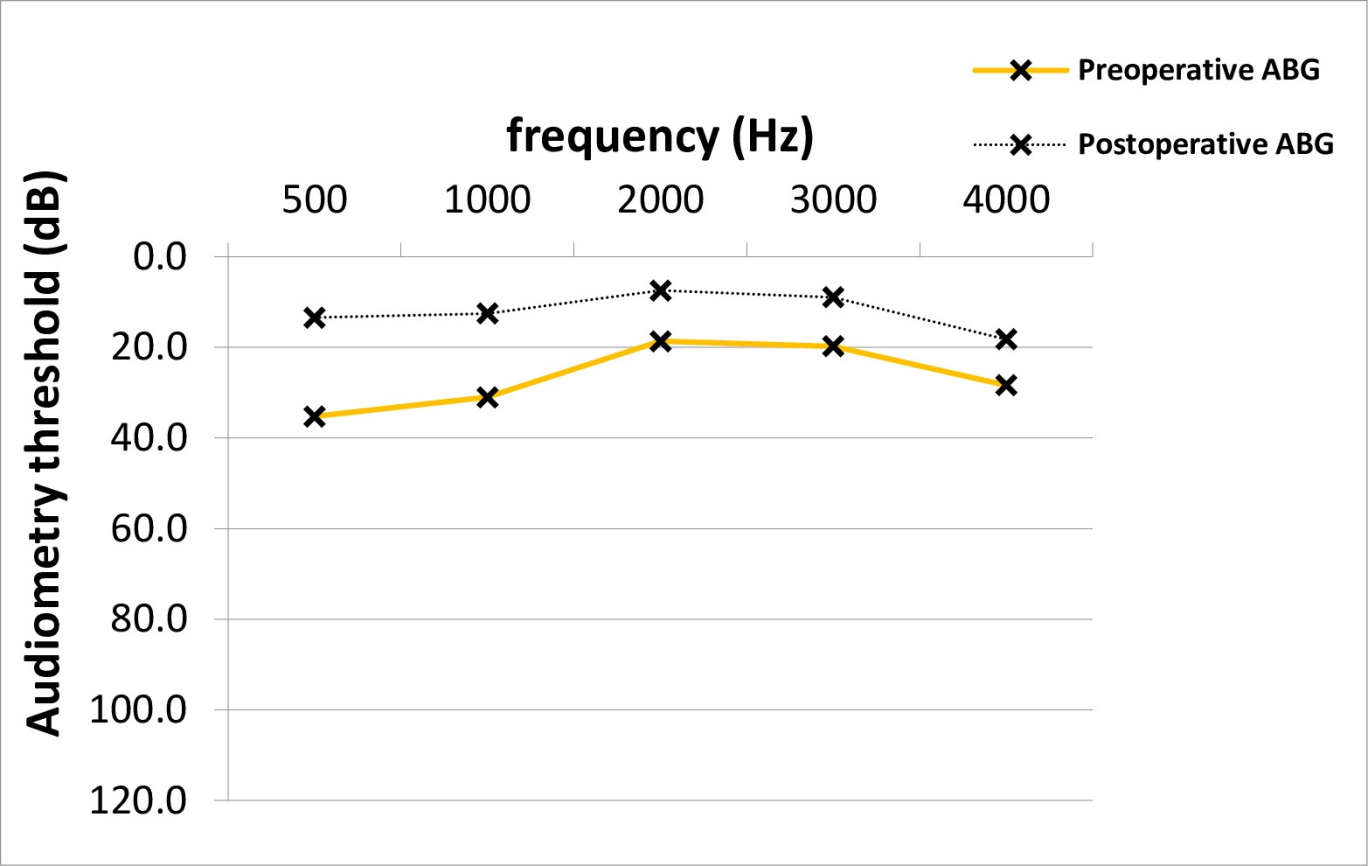
ABG closure across frequencies in PTA. The average postoperative ABG across frequencies was significantly lower than the average preoperative value (Paired T-test, p = 0.001). ABG; air-bone gap, PTA; pure-tone audiometry, dB; Decibel, Hz; Hertz.

**Fig 3:**
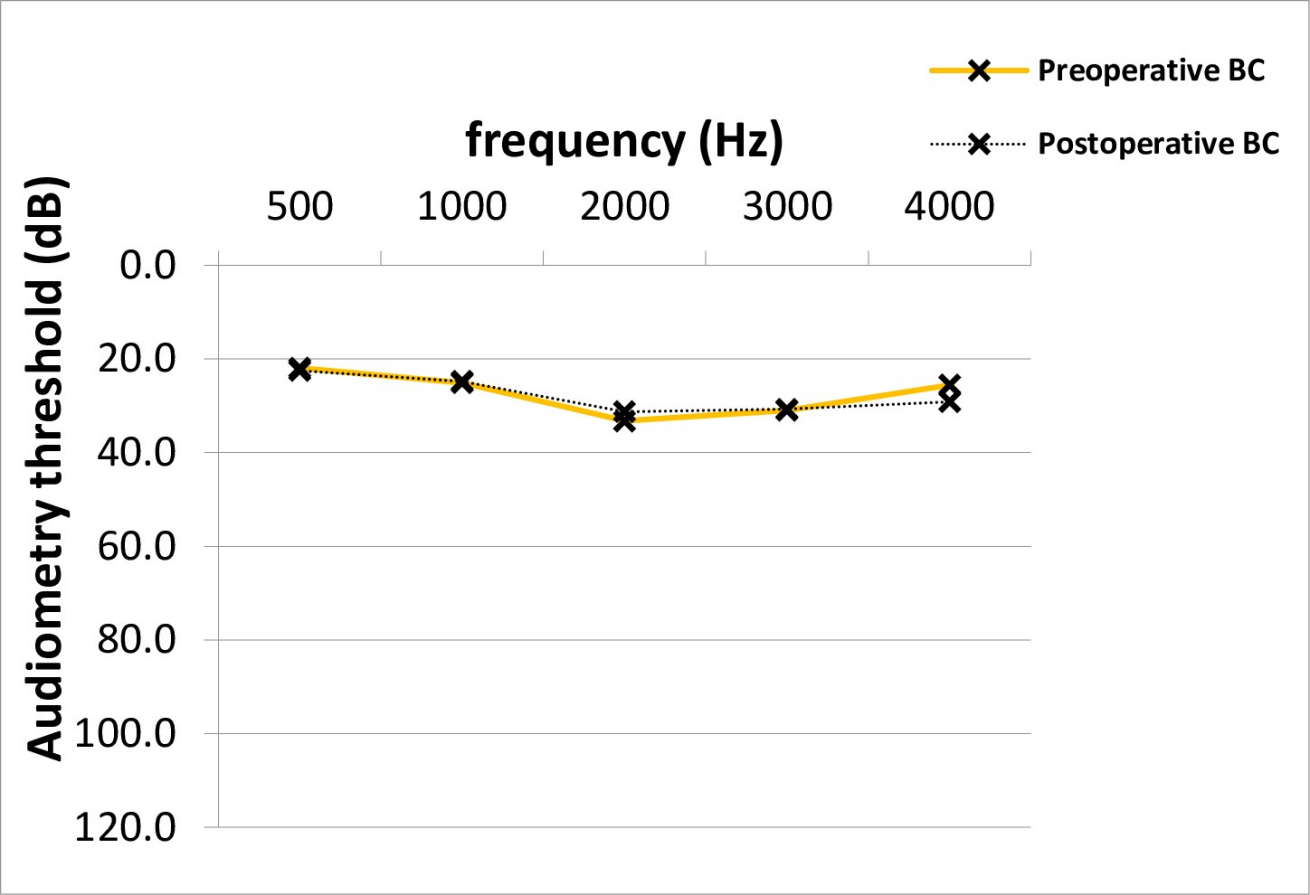
Preoperative and postoperative BC thresholds across frequencies in PTA. No significant difference between average pre- and postoperative BC PTA values across frequencies was shown (Paired T-test, p = 0.7). BC; bone-conduction, PTA; pure-tone audiometry, dB; Decibel, Hz; Hertz.

At time of stapes surgery, the mean AC thresholds across all frequencies were 53.9 dB and improved significantly after surgery to 39.8 dB (14.2 +/- 14.8 dB, p = 0.001; median 15.0 dB) (Fig 1). The preoperative average ABG was 26.5 dB and improved 12.0 dB after surgery (14.5 +/- 12.8 dB, p = 0.001; median 15.0 dB) (Fig 2). No clinically significant change was observed in average bone conduction (BC) thresholds (from 27.4 to 27.7 dB, p = 0.7) (Fig 3).

The average surgeon’s experience was significantly shorter in 50 cases with postoperative complications in the follow-up compared to other 617 procedures (4.9 vs 7.3 years, p = 0.001) performed stapes surgery. Yet, the surgeons´ experience did not correlate with AC improvement (p = 0.964) or with ABG closure (p = 0.682). Perioperative antibiotic prophylaxis was neither associated with AC improvement, ABG closure nor complication rate (p = 0.336, p = 0.603 and p = 0.517, respectively). In contrast, perioperative application of cortisone was associated with a lower complication rate (7.8% vs. 16.2%, p = 0.002) and AC improvement (14.9 +/- 14.1 dB vs. 10.2 +/- 18.9 dB, p = 0.016), but not with a better ABG closure (p = 0.398). The laser footplate perforation had significant positive effects on audiological outcomes (average ABG closure: 15.9 +/- 13.3 dB vs. 13.5 +/- 12.5 dB, p = 0.05; average AC improvement: 16.1 +/- 14.8 dB vs. 12.9 +/- 14.8 dB, p = 0.025) as well as on the postoperative complication-rate (5.3% vs. 11.1%, p = 0.017).

### Prognostic factors

A univariable and multivariable binary logistic regression model was performed in order to test whether different variables *i)* affect outcome and *ii)* represent independent prognosticators. Usage of CO_2_ laser was the sole independent prognostic factor for AC improvement (OR 2.53; p<0.001) and ABG closure (OR 1.77; p<0.001) (Tables 2 and 3). Intraoperative application of systemic cortisone lead to an almost 3-fold decrease of overall complications (OR 0.34; p = 0.001) (Table 4).

**Table 2:** Binary linear regression model for AC improvement.

AC Improvement	Univariable Analysis			Multivariable Analysis		
	OR	95% CI	p-value	OR	95% CI	p-value
Age (low vs. high)	1.08	0.7–1.6	0.700			
Sex (female vs. male)	1.33	0.9–2.0	0.201			
Antibiotic (yes vs. no)	1.12	0.8–1.7	0.563			
Cortisone (yes vs. no)	2.38	1.4–5.0	**0.002**	1.76	1.0–3.4	0.068
Laser (yes vs. no)	2.94	2.0–5.0	**<0.001**	2.50	1.7–3.3	**<0.001**
Experience (≤6 vs. >6 years)	0.74	0.5–1.1	0.127			
Prosthesis type	0.80	0.6–1.1	0.142			

AC; air-conduction, OR; odds-ratio, CI; confidence interval.

**Table 3:** Binary linear regression model for ABG closure.

ABG Closure	Univariable Analysis			Multivariable Analysis		
	OR	95% CI	p-value	OR	95% CI	p-value
Age (low vs. high)	0.94	0.6–1.4	0.777			
Sex (female vs. male)	1.4	0.9–2.2	0.125			
Antibiotic (yes vs. no)	2.02	0.7–1.4	0.923			
Cortisone (yes vs. no)	2.27	1.3–3.3	**0.003**	1.78	1.0–3.3	0.070
Laser (yes vs. no)	2.56	1.7–3.3	**<0.001**	2.22	1.4–3.3	**<0.001**
Experience (≤6 vs. >6 years)	0.81	0.5–1.2	0.291			
Prosthesis type	0.91	0.7–1.2	0.505			

AC; air-conduction, OR; odds-ratio, CI; confidence interval.

**Table 4:** Binary linear regression model for overall complication-rate.

All complications	Univariable Analysis			Multivariable Analysis		
	OR	95% CI	p-value	OR	95% CI	p-value
Age (low vs. high)	1.85	1.0–3.4	**0.043**	1,75	0.9–3.2	0.073
Sex (female vs. male)	0.52	0.3–0.9	**0.027**	0,56	0.3–1.0	0.062
Antibiotic (yes vs. no)	0.75	0.4–1.4	0.333			
Cortisone (yes vs. no)	0.32	0.2–0.6	**<0.001**	0.34	0.2–0.7	**0.001**
Laser (yes vs. no)	0.45	0.2–0.9	**0.036**	0.85	0.4–2.0	0.703
Experience (≤6 vs. >6 years)	2.00	1.1–3.7	**0.029**	1,49	0.8–2.9	0.242
Prosthesis type	0.92	0.6–2.4	0.672			

AC; air-conduction, OR; odds-ratio, CI; confidence interval.

Patients with 36.9 years or less had an independent higher risk for developing a postoperative conductive hearing loss. Moreover, a higher risk for postoperative hearing loss was observed in cases operated by surgeons with 6 or less years of experience (OR 5.1; p = 0.033) (Table 5). Notably, out of 308 cases operated by surgeons with more than 6 years of surgical experience, 15 (4.9%) developed a postoperative complication. Otherwise, out of of 359 surgeries performed by more experienced surgeons, 35 (9.7%) were associated with a complication in the follow-up.

**Table 5:** Binary linear regression model for persisting or recurrent conductive hearing loss.

Persisting or recurrent conductive hearing loss	Univariable Analysis			Multivariable Analysis		
	OR	95% CI	p-value	OR	95% CI	p-value
Age (low vs. high)	4.10	1.1–14.7	**0.030**	3.83	1.1–13.8	**0.040**
Sex (female vs. male)	0.71	0.2–2.3	0.568			
Antibiotic (yes vs. no)	0.55	0.2–1.7	0.258			
Cortisone (yes vs. no)	0.44	0.2–1.3	0.126			
Laser (yes vs. no)	0.76	0.2–2.5	0.649			
Experience (≤6 vs. >6 years)	5.47	1.2–24.4	**0.026**	5.13	1.1–23.0	**0.033**
Prosthesis type	0.87	0.4–1.8	0.707			

## Discussion

The current study provides a retrospective assessment of clinical outcome and postoperative complications after stapes surgery in a single center over a 27-year period. To the best of our knowledge, this is the first study, although of retrospective design, analyzing the impact of the surgeons’ experience and the perioperative administration of antibiotics on outcome in stapes surgery.

The average AC improvement was slightly lower compared to results in literature [6, 7]. However, the mean ABG closure and the postoperative complication-rate were similar to data reported by others [6–8]. In particular, the rate of postoperative sensorineural hearing loss (>10 dB) was comparable to the literature (1.7% [15] to 5% [16]). Regarding postoperative vertigo the incidence in the current literature ranges from 1.1% to 2.9% [2–7] and is in accordance with our data. Furthermore, the rate of postoperative wound infections was low whereas a slightly higher rate was noted in a large national register analysis [2]. Finally, eardrum perforations occurred significantly less frequently in our study group than reported by another author (0.1% vs. 2.6%) [7].

There are no studies exclusively reporting effects of perioperative single-shot antibiotic prophylaxis on outcome in stapes surgery. As already mentioned, only two reviews of non-recent literature could be found. These recommend no antibiotic prophylaxis in clean otology surgery [10, 11]. In regards to clean-contaminated ear surgery, contrary results are reported. For example, a study by Pierce et. Al [9] noted a lower rate of postoperative SSI in patients with contaminated cholesteatoma after tympanoplasty receiving single-shot antibiotic prophylaxis. In our patient cohort, only one case of SSI was observed. As there is only limited and outdated data in literature, up to now a single-shot antibiotic was administered in many of stapes surgery cases in our department. However, risks of intravenous antibiotics such as an allergic reaction are moderate [17]. We observed no benefit of perioperative antibiotic prophylaxis on surgical or audiological outcomes. Therefore, the conclusion can be drawn that optimal prevention of SSI in stapes surgery consists of proper preparation and disinfection of the surgical area without need for antibiotic prophylaxis.

The surgeons experience has shown to be associated with favorable outcome in urology [12], maxillofacial surgery [13], neurosurgery [14] and even when performing a simple fine-needle biopsy [18]. Interestingly, there is only one study showing a significant association between surgeons´ experience and outcome in ear surgery. In particular, 400 tympanoplasties performed by the same surgeon showed a better outcome with increasing experience [19]. To the best of our knowledge, our study is the first assessing the experience of the surgeon on the outcome in stapes surgery. Six or less years of surgical experience are an independent marker for an increased rate of a persisting or recurrent postoperative conductive hearing loss. According to the intraoperative findings of surgically revised cases, hearing loss was caused either by prosthesis luxation or incus necrosis. The crimping of the prosthesis loop onto the long crus of incus is considered being the most delicate maneuver in ear surgery. It has been noted that malcrimping of the prosthesis is a risk factor for prosthesis luxation [20] and for necrosis of the long crus [21]. Hypothetically, less experienced surgeons could possibly have difficulties when crimping the prosthesis that potentially led to subsequent dislocation of the prosthesis or to necrosis of the long crus revealing as a conductive hearing loss later in the follow-up. Therefore, we underline the necessity of a well-founded surgical training in stapes surgery as well as the need for high sense of responsibility of mentors for their trainees and for longer periods of supervised operations. Appropriate training for residents and prolonged surgical supervision of young otology surgeons is of tremendous importance. It seems that giving particular attention to proper teaching of the crucial surgical steps such as crimping of the prosthesis might be essential in lowering the complication-rate in later unsupervised surgery. We therefore conclude that this should be one of the focal points of surgical training in stapes surgery.

Surgical experience can be assessed differently (number of procedures performed, years of experience or even by level of expertise e.g. by comparing training to attending physicians). We decided to analyze it solely as years of experience due to several considerations. First, some surgeons perform surgeries in other centers (data on these could not be retrieved nor included in the analysis). Second, from the time of first surgery, in our center the attention is given that each surgeon performs similar number of surgeries. Therefore, on average, similar number of surgeries can be assumed on a yearly basis for every physician. Lastly, not just exclusively performing surgeries contributes to the surgeon’s experience. Factors in the medical and surgical environment (such as observing time spent in the operating room or gathering experience by spending time in the outpatient department) could contribute substantially to surgical experience (decision making, setting the right indication, deciding on the postoperative handling etc.).

Other factors influencing clinical outcome after stapes surgery are perioperative administration of corticosteroids and the use of CO_2_ laser for footplate perforation. In particular, anti-inflammatory effects of corticosteroids could inhibit soft-tissue reaction and the formation of the periprosthetic fibrosis and should therefore lead to better outcomes [22, 23]. In our patient cohort, the use of intravenous perioperative cortisone had an independent positive impact on the overall complication rate. Notably, in the univariable analysis cortisone administration was associated significantly with the average AC improvement but not with the mean ABG closure. This was caused by different postoperative BC thresholds. Besides BC thresholds deteriorating as a possible complication in ear surgery, a paradoxal postoperative BC improvement can occur after stapes surgery. Interestingly, increased postoperative BC thresholds were reported by Lavy et al. [24] in 44% of patients undergoing a stapedotomy. This study group even raised the question if preoperative BC thresholds could be accurately measured in otosclerosis due to the Carhart effect. Even the American Academy of Otolaryngology, Head and Neck Surgery suggests adding 10 dB to preoperative BC thresholds when assessing the ABG in order to represent the Carhart effect [24].

Perforating the footplate with the laser may minimize the mechanical manipulation and damage. This could contribute to firmer fixation of the prosthesis and better short-term as well as long-term results. Favorable outcomes of laser stapes surgery when compared to manual perforation were already published [25, 26]. In the current study, the use of laser for footplate perforation was independently associated with better audiological outcomes.

As this was a retrospective chart analysis, some standard limitations for this type of studies could have played a role. First, some data could not be retrievable, particularly from the earlier years before digitalization of the hospital database system. Furthermore, when comparing outcome between different cohorts, no proper matching between groups could be performed retrospectively. Finally, confusion bias regarding confounding factors can hardly be avoided. However, these limitations were minimized and the clinical relevance and significance of the results could be emphasized by the large patient sample.

## Conclusion

In conclusion, present data show that surgeons’ experience was linked to better clinical outcome in stapes surgery. Postoperative conductive hearing loss caused by incus necrosis and prosthesis luxation was less often observed in surgeries performed by physicians with more than 6 years of experience. This underlines the paramount importance of an adequate surgical training in stapes surgery. Furthermore, the current study does not support the use of perioperative antibiotic prophylaxis in stapes surgery. Finally, analysis shows a better audiological outcome in surgeries performed with the CO_2_ laser.
